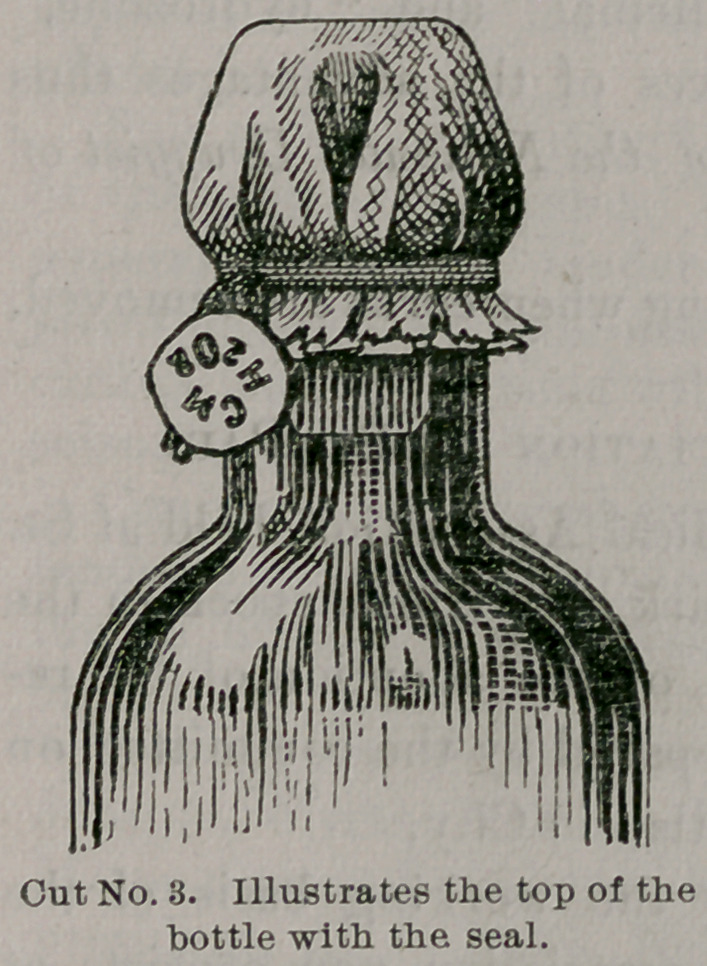# Selections and Abstracts

**Published:** 1901-07

**Authors:** 


### SELECTIONS AND ABSTRACTS.

### The Role of the Infections in Diseases of Women.*

The rôle of the infections in diseases of women, in view of the importance of the subject, should be accorded more distinct recognition than is, at present, given it, in either the periodical or the text-book literature. This importance becomes more and more apparent, as we emancipate ourselves from the old conceptions of the etiology and pathology of the so-called inflammatory diseases of the female genitalia. The influence of a nomenclature in determining and limiting the understanding of a subject was never more clearly demonstrated than in the employment of terms used to describe various clinical and pathological conditions that are constantly brought to the attention of the practitioner of gynecology. Thus, we hear of "vaginitis" and "endocervicitis" and "endometritis" and "salpingitis" and "ovaritis" and "puerperal fever," terms which signify certain more or less indefinite disturbances of the circulation in the various anatomical structures to which they obviously apply; while, as a matter of fact, no one of these terms conveys or can convey, if accepted in the light of their usual respective definitions as indicating anything like an accurate picture of the conditions which they are each expected to describe. The time was when inflammation was accepted as a pathological entity, a local nutrient disturbance occurring *de novo*, and terminating spontaneously in either resolution or suppuration. This conception still persists to an important degree in the professional mind, with the result that relatively too little attention is given and too little importance is attached to those phenomena which are necessarily antecedent and consecutive to inflammation proper. The evil resulting from this misconception consists in a failure to consider, at their true value, on the one hand, measures of prophy-

*Read by Charles A. L. Reed, M.D., President of the American Medical Association, Cincinnati, Ohio, before the Medical Society of the State of New York at Albany, January 30, 1901.

laxis, and, on the other, necessary means of cure. It is with the object of impressing anew the character of the conditions antecedent to inflammation, as manifested in gynecologic practice, that this paper is presented.

At the outset, I wish to urge the view that the essential antecedent condition of inflammation of any part of the female genital tract, or of inflammation elsewhere, is that of infection.

### THE NATURE OF INFECTION.

It is not assumed that infection must exist exclusively as, in fact, it does generally, of pathogenic micro-organisms, for we find that the toxins which they generate are among the most serious of the disease-producing agents; and as between the toxins of micro-organic origin and the toxins of vegetable or of mineral origin, it is not necessary at this juncture, and for our present purpose, to attempt any discrimination.

I wish to go a step further and affirm that without infection, as here defined, inflammation is not demonstrable. It is not sufficient to urge as an exception to this rule, and one calculated to break its force, that all the circulatory phenomena of inflammation can be induced by irritating substances, such, for example, as croton oil. On the contrary, this very alleged exception, is only another testimony of the universality of the law; for these substances, croton oil in particular, are only so many infectious elements against which the phenomena of inflammation are manifested, as barriers against systemic invasion. "Infection" must be recognized as the generic term towards which "intoxication" sustains a subordinate relation.

With this understanding of the subject, we are enabled to comprehend that, except in examples of repair following traumatism, either surgical or otherwise, the presence of inflammation necessarily implies the pre-existence of infection, and we are at once confronted with the natural inquiries: (1) What is the character of the infection? (2) What are the natural barriers against destructive invasion of the genitalia by infectious elements? (3) What are the natural means by which infectious elements, once introduced, are restricted in their activity to a more or less definite locus?

(4) What are the means and influences by which the vitality and virulence of pathogenic micro-organisms are limited ?

### BACTERIA OF THE GENITALIA.

The possibility of infection is always present in the female genital organs. This fact is made more conclusive by the constant existence of micro-organisms, many of them pathogenic in character, on the cutaneous surfaces of the vulva and on the vaginal mucous membrane, in a state of health; although, normally, the genital canal is germ-free above the *os uteri externum.* The bacteria found upon the vulva in health are generally saprophytes of but little importance, but with them have been found the streptococcus pyogenes, the staphylococcus pyogenes aureus, the staphylococcus epidermidis albus and a micrococcus feditus. These, among other micro-organisms, have been found in this locus unattended by any manifestation of disease, a circumstance which would indicate that, so far as these bacteria are concerned, the healthy vulva is, to state it negatively, not an unnatural habitat.

The bacteriology of the vagina in health has been a subject of patient research, with the result that it is now accepted as true that in normal states the vagina contains no pathogenic aerobic bacteria, although it abounds in organisms of the anaerobic variety, among which may be mentioned two special varieties, namely: (1) the micrococcus feditus of Veillon, which increases in numbers relatively to the distance from the introitus; and (2) the bacillus vaginalis of Doederlein, which is a recognized acid-former, and which, like the preceding, is more abundant, near the uterus, than near the vaginal outlet. Various varieties of staphylococci and streptococci, have also been found in the symptomatically normal vagina; but these, unlike the preceding, exist in greater numbers near the introitus, than near the uterine extremity of the canal.

### NATURAL BARRIERS AGAINST INFECTION.

The question naturally arises in this connection, why do not these micro-organisms, existing on the vulva and in the vagina, always produce phenomena of disease. The answer must be more or less theoretical. The first explanation, and one which is not theoretical, is that the epithelium in its integrity is a sufficient barrier against the invasion of underlying structures by disease-producing bacteria. The next explanation is that the virulence of pathogenic bacteria is minimized, if not destroyed, by the secretions found in the vagina, although the bactericidal element of these secretions is not yet definitely determined. It has been assumed, with some show of truth, that the acid-forming bacillus of Doederlein, indigenous to the vagina, exercises an important office in this connection. The question of virulence, however, and the influences by which it is modified, is one which cannot be answered in the light of our present knowledge, by anything like positive assertion. The absence of air, or more properly of oxygen from the vagina, determines the fate of aerobic bacteria. Those of the anaerobic variety, however, would seem to be debilitated, if not destroyed, by the influence of the toxic products of the bacillus vaginalis of Doederlein, and by the influence of the secretion, alkaline in character, from the cervical canal. It is to this secretion that much importance is to be attached in limiting the propagation of bacteria to the vagina in conditions of health. Not only its chemical properties, but its physical qualities, its viscidity, and the direction of its current, are effective means of preventing the spread upwards of organisms not possessed of strong ameboid activity. The barrier thus formed is fortified by the peculiar anatomical confirmation of the cervix, its margins, and the plicæ of its lining membrane. We may, therefore, summarize by stating that normal invasion of the genitalia by micro-organisms is limited to the intra-uterine segment of the tract by the epithelium, by the character of the vaginal and cervical secretions and by the peculiar construction of the cervix.

### CONDITIONS FAVORABLE TO PATHOGENICITY OF BACTERIA.

If, however, the secretions of the genital tract are studied in the presence of the so-called inflammation, it will be discovered that the same micro-organisms which have been found on the vulva and in the vagina in health, now exist in those localities, and possibly higher up, in a state of increased virulence. Why were they innocuous before, and why are they actively pathogenic now? The answer must be found in the assumption of conditions favorable to their propagation and to their increased power for mischief. It is true that the expression "favorable condition" is somewhat ambiguous, but it assumes a more definite significance when those conditions are subjected to analysis. In the first place, it is generally discovered that the already mixed flora of the vulva and vagina has received fresh accessions. The most frequent accession is the gonococcus of Neisser, which, in this locus, manifests its acme of virulence. It assails the epithelium; it overcomes in large measure the antagonistic chemical properties of the secretion, and passes the barrier offered by the cervix. It thus invades in its triumphal way the contiguous epithelial surfaces until it has traversed the vagina, the endometrium and the endosalpinx. It may even advance until it reaches the surface of the ovaries and attacks the peritoneum. In its career of invasion, by overcoming the conditions which acted as insuperable obstacles to the preexisting vaginal bacteria, it furnishes safe escort to the latter and the case in its finality reveals itself as one of mixed infection.

Another example of modifying influence is to be found in traumatism, by which the protective or rather, the resistant power of the epithelium is destroyed, through the formation of an infection atrium. By this means, the streptococcus conveniently existing in the vagina may be carried by a tent or a sound or a dirty finger, from what may be recognized as its original habitat, to an entirely new and more congenial environment in the sub-epithelial tissues. Here, apparently in response to the stimulus derived from its new surroundings, it propagates rapidly and establishes destructive processes in the tissues themselves; or the organisms themselves or their toxins, "coursing through the natural gates and alleys of the human system," manifest their intense and most disastrous enmity to the blood of man.

THE STATUS OF INFLAMMATION IN THE PROCESS OF INFECTION.

The effort of nature to limit an infection to the point of primary invasion is manifested by sudden and marked disturbances in the local circulation. There occurs a dilatation of the capillaries, quickened circulation, which is speedily followed by a lessened velocity and final stagnation of the blood current; and, what is most important of all, a rapid multiplication and migration of the leucocytes. The transuded liquor sanguinis furnishes the medium in which these corpuscles exercise their phagocytic activity in repelling the invasion by the pathogenic micro-organisms. The phenomena to which I have just alluded will be recognized as those which from time immemorial have been given in the classical descriptions of inflammations. It will thus be seen that inflammation, whether considered in its acute form, as just presented, or whether contemplated in its more chronic aspects of resorption and organization, must be recognized as a step, and only an intermediate step, in the general process of infection. It is of highest importance, therefore, that in considering any of the so-called inflammations we take into account not only a part but all of the pathologic process. To this end, it is manifestly better to speak of these cases as infections, rather than as inflammations. As additional reasons for revising our nosology, in accordance with this view, it is urged that different infections produce different conditions and different symptoms, and demand, consequently, different treatment.

INDIVIDUAL INFECTIONS.

It may be urged in opposition to this view that, as already stated, infections of the female genitalia, and systematic infections depending thereon, generally depend upon more than one active agent; or, in other words, that these infections are generally of the mixed type. As an absolute fact, this is true; yet, it is equally true, almost without exception, that infection in any given case is generally dominated by some particular micro-organism. This is exemplified in puerperal fever, in which the essential factor is the staphlycoccus pyogenes; but in which numerous other pathogenic bacteria are demonstrable, either at the site of the primary invasion, or in the secondary suppurations; yet, the clinical phenomena are essentially and distinctly those of streptococcic infection. In a case of pyosalpinx, the result of progressive invasion of epithelial surfaces by the gonococcus, the pus contained in the Fallopian tube may abound in staphylococci, yet the history of the case, its symptoms and its pathology are distinctly those of gonococcic infection. It is needless to speak of diphtheria, thrush, syphilis, or other distinct infections, in which there occurs in a distinctly subordinate and unimportant degree more or less of mixed infection. These considerations are presented with the hope that more careful study will be given the rôle of the different infections, and that in the not distant future the diagnosis of these cases may be based upon the essential etiologic factor, rather than upon an intermediate and distinctly incidental pathologic process.

In this connection it is of importance that our knowledge has so far advanced that we may recognize, not alone pathologically but clinically, a number of the individual infections. I do not now allude to the symptomless presence of pathological bacteria in the external genitalia but to these same, or other bacteria in a state of virulent activity. In some instances this activity is manifested in one part, and, in others, in another part of the genital tract. Thus gonococcic infection may manifest its destructive activity either upon the epithelial surfaces of the entire genital tract, or finding an infection atrium, it may traverse the circulatory media, occasioning either systemic intoxication or local suppuration within or even beyond the pelvis. The tendency of this micro-organism to advance upon the mucous surfaces and its power, in doing so, to overcome the natural barriers to invasion makes it the most destructive of all the individual infections of the female genitalia. The streptococcus pyogenes which has been demonstrated to exist in the symptomatically healthy vagina may likewise become the element of a diffuse infection which, attacking the deeper structures of the genitalia, may manifest its virulence either in the destruction of the tissues with which it comes in contact, or, reaching the blood stream, produce the phenomena of pyemia. It is not, however, within the proper limits of a paper such as this to sketch, even thus briefly, the course of the various individual infections, such as that due preponderatingly to either of the various staphylococci, to the bacillus tuberculosis, to the pneumococcus, to the bacillus coli communis, to the bacillus aerogenes capsulatus, to the actinomyces, to the septic vibrion or to the echinococci. It is hardly necessary to mention such additional examples of infection as diphtheria, aphthæ (oidium albicans) and bilharzia, the micro-organism of each of which condition is comparatively well understood, nor is it necessary to speak of syphilis and chancroid, the infectious elements of which have not yet been isolated.

### CONSEQUENCES OF INFECTIONS.

The consequences of infections are various. Some, notably that due to either the gonococcus, or to the less virile streptococcus, or to the staphylococcus, may be limited to the vagina through the self-disinfecting power of that canal, although reliance upon this possibility is fraught with danger. In those cases in which the infection has become more general but is restricted to the genitalia, the resulting organic changes vary somewhat according to the predominating micro-organisms. Thus the tissue changes consequent upon invasion by the gonococcus are essentially different from those caused by the bacillus aerogenes capsulatus, or by the more familiar streptococcus pyogenes. It is true that in some of these infections death of the patient may come early in the process, or it may be delayed until often numerous changes have taken place, or it may not occur at all. But, without considering this contingency, we may recognize the organic changes consecutive upon infection as (a) follicular, manifested for the most part by more or less permanent hypertrophies; or (b) interstitial, manifested by the deposition or possibly the more or less distinct organization of inflammatory products; or (c) in the destruction of the superficial epithelium and the agglutination of opposing mucous surfaces; or (d) in the organization of plastic exudates on serous surfaces. The first of these processes is exemplified in the hypertrophy of the mucous follicles of the vagina and the endometrium, and of the vulvo-vaginal glands; the second in the more or less permanent induration of the cellular structures following an infection, and the third in the sealing of the Fallopian tubes as revealed in every case of pyosalpinx. While the last named process, namely that of organization of plastic exudates on serous surfaces, is shown in the development of an adventitious tunic upon the surfaces of the infected ovary, and in the development of adhesions between proximal peritoneal surfaces wherever they have been the seat of infection. The functional results are not less striking. Ovulation is made painful, menstruation becomes distressing, coition becomes annoying, and in a majority of instances sterility is the result. In the meantime the general health yields to the constant source of annoyance and distress, and the patient joins the ranks of the chronic invalids.

### DIAGNOSIS OF THE INFECTIONS.

It is obvious that diagnosis of the infections is of very great importance, and I mention it in this connection only to state that the usual methods of examination must be supplemented with those of greater precision. The microscopic examination of infected secretions is imperative, and the revelations of the microscope should be supplemented with culture and inoculation experiments when necessary. Practically all of the morbid states within the pelvis have a more or less direct bearing upon the hematogenetic function, and the more careful study of the blood is destined to yield more precise data than we yet enjoy, upon which the diagnosis of intrapelvic states may be predicated.

### THE LIMITATION OF INFECTIONS.

The practical importance of these views has already found expression in the now well-evolved principles of antisepsis as applied to both gynecologic and midwifery practice. The known presence of pathogenic bacteria in the vagina has led to the careful sterilization of that canal before operations are done, either upon it or upon the uterus, and before parturition. It would be manifestly improper for me, elementary as is this paper, to dwell further upon these details. I cannot resist the temptation, however, to call attention to some additional applications of the principles which I have enunciated. Permit me to take a familiar example: laceration of the cervix results necessarily in an enlargement of the uterine ostium. The condition is generally associated with follicular hypertrophy, and an exaggerated secretion of thick tenacious mucus. It must be recognized at a glance that this change in the size of the uterine orifice and in the lumen of the cervical canal favors the invasion of the uterus by infectious elements residing in the vagina. The tendency thus established is counterbalanced to a certain extent by what may be recognized as a compensatory increase of the cervical secretion. This secretion by virtue of its increased volume, its natural viscidity, its equally natural alkalinity and the course of its current is practically the only remaining barrier to the infection of the endometrium. Treatment directed to such a case should not be limited to a currettage, and to the cure by other means of the follicular hypertrophy, for, in the event of success, it would only remove an important safeguard. It becomes obvious, at a glance, that treatment in such a case should never stop short of restoration of the cervix. We thus see that the operation of Emmet has an additional claim for consideration, and I have no hesitancy in urging that one of the strongest reasons why a cervix even slightly lacerated should be repaired is the prevention of probable infection of the endometrium, and secondarily of the Fallopian tubes.

### THE PROPHYLACTIC TREATMENT OF INFECTIONS.

But manifestly the practical point emphasized by the preceding considerations must be the disinfection of the vagina. It may be premised in this connection that the vagina does possess to a certain degree the power of self-disinfection; yet, as demonstrated by numerous observers, and as already stated in this paper, pathogenic bacteria are frequently found in that canal. It follows, therefore, that in these cases the self-disinfecting power of the vagina is not sufficient to remove the dangers that lurk in the folds of that canal. It would seem that where these conditions exist, art can add but little to the resources of nature. The systemic experiments of Steffeck (Bacteriologische Begrundung der Selbstinfection, 1890) to disinfect the vagina by mechanical and other means were attended with only partial success. Menge endeavored to eliminate the streptococcus pyogenes from a vagina in which it had become an inhabitant. The walls of the canal were treated with detergents, were vigorously scrubbed, and subsequently irrigated with strong solutions of carbolic acid, and of corrosive sublimate, in different degrees of concentration. He also subjected his patient to long treatment with lactic acid. The result of his efforts and his nearest attainment of success was to reduce the infection for only a short time to isolated colonies of streptococci, which immediately began to remultiply, the original condition being reproduced in from three to six hours. He finally succeeded in eliminating the streptococcus by the application of a 50 per cent. solution of zinc chloride, a remedy too severe to be thought of for ordinary practice, but after its employment the streptococcus was displaced by a short anaerobic bacillus. It is evident from these observations, which have been confirmed in a practical way by many practitioners, that the disinfection of the vagina when once invaded by pathogenic bacteria is exceedingly difficult. In the majority of these cases, as emphasized by Menge, it becomes necessary after all to rely upon the self-disinfecting power of the canal, and this power, as pointed out by St. Clair, works only against the organisms that are at once true parasites and facultative aerobes.

The broader question of prophylaxis relates not alone to aseptic precautions in manipulation within the vagina and uterus, but to the limitation of the infection of venereal origin. This is a sociologic question of primary importance. On this point, I ask to be pardoned for reiterating views that I have expressed elsewhere (Text Book of Gynecology), as follows:

"The social evil being recognized as a fixed and inevitable fact, and the dissemination through it of venereal disease being so destructive to women, it is the manifest duty of society to subject prostitution to the most rigorous supervision. The medical profession owes it to itself, and to the humane objects to which it stands consecrated, to use its influence to secure the legal regulation of that evil which society has proved itself unable to suppress."

### CONCLUSIONS.

I concluding this paper, I beg leave to formulate tentatively at least a few of the laws which seem to determine the rôle of the infections in diseases of women,

1. The epithelial surface of the genital tract in its integrity is an efficient barrier against invasion of the underlying structures by pathogenic micro-organisms that establish parasitic and saprophytic relations to the vagina.

2. The normal cervix and its contained secretions are adequate barriers against invasion of the uterus by pathogenic bacteria that are capable of maintaining a habitat in the vagina.

3. The vagina possesses certain powers of self-disinfection, which work only against the organisms that are at once true parasites and facultative aerobes.

4. Certain pathogenic bacteria, notably the gonococcus of Neisser, the Klebs-Loeffler bacillus and the oidium albicans, find in the warmth and moisture of the genital epithelium, conditions favorable to their propagation and to the increase of their virulence whereby the epithelium itself may be destroyed, to the extent of losing its protective properties.

5. Pathogenic bacteria innocuously present in the genital tract may become virulent when introduced into the underlying structure through a breach in the protective epithelium.

6. Pathogenic bacteria, when introduced into previously normal tissues, immediately provoke the process called inflammation, the essential phenomena of which is the speedy deposit and rapid extravascular migration of the leucocytes, which act as phagocytes in preventing the further invasion of the system.

7. Pathogenic bacteria that are not thus overcome by the leucocytes may enter either the lymphatic or the sanguiniferous circulation, producing secondary phenomena, septicemia, pyemia, and even the death of the patient.—*St. Paul Medical Journal.*

---

### THE ALUM ENEMA IN THE AFTER-TREATMENT OF ABDOMINAL OPERATIONS.*

The improvements introduced into the technique of abdominal work during the last few years have largely eliminated the dangers which formerly characterized that department of surgery. Rigid antisepsis and thorough toilet of the peritoneum have materially diminished the mortality from septicemia, complete operation and new methods of hemostasis have reduced the danger of hemorrhage, while rapid operating and saline infusions have made fatal shock almost a thing of the past.

---

*Read by Virgil O. Hardon, M.D., Atlanta, Ga., at the annual meeting of the Medical Association of Georgia, April 17, 1901.

But there is one complication in abdominal surgery which has not kept pace with the general improvement and which claims nearly as large a percentage of victims to-day as it did ten or fifteen years ago. I refer to intestinal paresis. Every one who has done any considerable amount of abdominal work is familiar with this much-dreaded complication and knows how great a fatality characterizes it. It follows the simplest as well as the gravest operations, and when once it has become established the prognosis, under accepted methods of treatment, is in the highest degree unfavorable.

The best authorities are agreed that intestinal paresis following abdominal section is due to exudative peritonitis. It is not easy to believe that such is the case, in view of the fact, upon which all operators are also agreed, that free movement of the bowels causes the symptons to disappear in a few hours, and that when this is accomplished the patient goes on to a rapid recovery. Yet the reports of autopsies made after death from this cause compel an acceptance of that view.

Dudley has given a graphic description of the clinical history of such cases. He says: "Every abdominal surgeon is painfully familiar with the characteristic symptoms. He has descried them from afar as one may discern the black cloud near the horizon. In the balance between hope and fear he has watched the anxious face, the drawn expression, the progressively rising temperature; the nausea, at first attributed to the anesthesia, then, as this subsides, the vomiting of sepsis which takes its place; the frequent regurgitation of bile mixed with blood and mucus, and growing darker and darker. He has realized the gradual failure of the pulse, first weak, then running, then thready to the vanishing point; the paretic and distended bowels, which refuse to act; the rapid respirations, the cold extremities, the staring eyes, the wide nostrils, and finally the inevitable collapse." "Treatment," he adds, "is utterly useless. . . . The first effort should be directed to the movement of the bowels." With reference to the same condition, Penrose says: "If these symptoms are not arrested by the use of purgatives, turpentine enemata, and the rectal tube, it is probable the result will be fatal." Similar quotations could be given from many other writers on the subject, and the lurid picture thus drawn confirms the experience of every operator that the condition is one of the greatest gravity and one which calls for prompt and energetic treatment. All remedial measures which do not include thorough movement of the bowels are absolutely futile, and if this end be accomplished no other remedy is needed. The reestablishment of intestinal peristalsis is, therefore, the essence of treatment. It is not necessary that there should be a copious discharge of fecal matter. If peristaltic action be induced to the extent of causing expulsion of the gas by which the bowels are distended, the desired result will be accomplished and fecal discharges will follow later. This fact renders it highly probable that distention of the bowels by gas is the cause as well as an effect of the paralysis of muscular action in the intestinal walls.

Epsom salts has been found to be the best internal remedy for exciting peristalsis under these circumstances. But, unfortunately, in the great majority of instances vomiting is so prominent a symptom that this remedy is not available. The stomach rejects it as soon as it is taken. It is also true that the vomiting is most persistent and uncontrollable in just those cases in which catharsis is most urgently called for. What is true of salts in this respect is equally true of all other internal medication, so that the stomach cannot be utilized as an avenue through which to attack the disease. Catharsis by hypodermatic medication has been often attempted, but has always proved a failure. The rectum alone remains as a channel through which remedies can be introduced into the system. The ordinary enemata of soap and water, oxgall, turpentine, and other substances are sometimes effectual at the beginning of the abdominal distention. But after the symptoms have become well pronounced, these remedies usually fail to have any effect and are frequently retained, thus adding to the distention already present. The rectal tube carried above the sigmoid flexure, even to the ascending colon, will sometimes allow of the passage of a portion of the gas. But the absence of contractile power in the muscular walls of the bowels, and the presence of gas in the small as well as in the large intestine, often render this procedure useless. Attempts to draw off the gas by aspiration through the abdominal walls have proved futile, and at the present day are mentioned only to be condemned. When all these measures fail, as they will fail in a large percentage of cases, the patient goes from bad to worse, and death soon closes the mournful scene. These facts, which are patent to all abdominal surgeons, furnish my reason for offering to the profession for such cases a plan of treatment which has proved so satisfactory in my hands that it has entirely superseded all others. I refer to the alum enema.

Almost exactly nine years ago, on April 21, 1892, I operated on a patient for pyosalpinx, removing the appendages on both sides. The operation presented no unusual complications. There followed the ordinary ether nausea, but instead of subsiding in a few hours, as it commonly does, it continued through the next day. At the end of twenty-four hours there was evident distention of the bowels by gas. The temperature, which up to that time had remained below 100°, rose to 102°. The pulse ran up to 130 and began to be thready in character. I recognized the necessity for prompt action and prescribed Epsom salts, which was not retained. Calomel in half-grain doses every hour was also rejected by the stomach. Enemata of soap and water, castor oil, glycerin, turpentine, and ox-gall were successively used, but without avail. A rectal tube was passed high up into the colon, but no gas escaped through it. At the end of the second twenty-four hours the patient's condition was alarming, and, from my previous experience with intestinal paresis, I felt that the case was practically hopeless. In casting about in my mind for some means of relief, it occurred to me that, by analogy with the violent gastric peristalsis effected by alum when taken into the stomach, a similar effect might follow its injection into the bowel. I therefore directed the nurse to prepare a solution of an ounce of powdered alum in a quart of warm water and to inject it into the rectum, while I sat by and awaited the result. In about ten minutes I was rewarded by hearing the sound, so grateful to the ears of the abdominal surgeon, of flatus escaping from the rectum. A large volume of gas was expelled, and the patient was correspondingly relieved. In an hour the enema was repeated with a similar result. From that time on the gas was expelled at intervals spontaneously, the pulse increased in strength and diminished in frequency, the temperature fell rapidly until it was below 100°. The patient was practically convalescent on the following day, made a rapid recovery, and left her bed at the end of two weeks.

Since that time I have used the alum enema in hundreds of cases, and always with equally good results. I have not varied from the formula which I used in the first instance; but one of my assistants, on one occasion, misunderstanding my directions, used an enema of one half that strength with apparently as good results. But as no bad effects follow the use of the stronger solution, I still use an ounce of powdered alum to a quart of warm water, which is approximately a three per cent solution. It usually causes expulsion of gas in from five to fifteen minutes, but in some cases a longer time is required. Sometimes it is necessary to repeat the injection before it will act. This can be done with perfect safety an indefinite number of times. There may be a reaccumulation of gas after the first enema has done its work. If so, the injection may be repeated as often as the gas accumulates. There is sometimes some pain attending its use, but it is not severe. It is not necessary that the solution should be carried high up in the colon. I inject it in the same manner that I would an ordinary enema, and probably in no instance does it go above the sigmoid flexure. But peristalsis is induced throughout the whole intestinal tract, including the small as well as the large intestine. In a certain proportion of cases in which the alum enema has been repeated several times, there is thrown off from the bowel a tubular cast. The first time that this occurred I was much alarmed, as I feared that it was due to sloughing of the mucous coat of the intestine. But a microscopic examination made by Dr. H. C. Moncrief, house surgeon of St. Joseph's Infirmary, showed that the cast was composed simply of mucus whose albuminous elements had been coagulated by the alum. I have therefore come to regard this phenomenon as of no importance.

As to the manner in which the alum enema acts in producing movement of the bowels, I have no theory to offer. That it does not act mechanically by its bulk is shown by the fact that in cases in which a similar quantity of water or of any other fluid has been injected without any result the alum solution is effectual. It seems to have as specific an action in inducing intestinal peristalsis as has castor oil when taken into the stomach. In only a small proportion of cases is the peristalsis sufficiently violent to produce griping pains. It does not cause a serous exudation from the intestinal walls, as does Epsom salts, and therefore produces less depletion of the abdominal blood-vessels. For that reason I prefer to use Epsom salts when the stomach will retain it.

The advantage which I claim for the alum enema consists in the promptness and certainty of its action in a class of cases in which all other remedies frequently fail, and in which such failure involves the death of the patient. It is, therefore, a life-saving measure, and as such I feel it my duty to offer it to the profession.

It has fallen to my lot to do abdominal work for the last sixteen years. During the first seven years, in which I did not use the alum enema, 42 per cent. of my deaths were due to, or at least accompanied by, intestinal paresis. During the last nine years, in which I have used the alum enema, I have not had a single death from that cause. I have seen only one patient in whom the alum enema failed to move the bowels promptly. In that case the enema was used every two hours during the third day without any apparent effect, and it was therefore discontinued. But the bowels acted spontaneously on the fourth day and the patient recovered. This may have been a case where the action of the alum was delayed for a longer period than usual. During the nine years in which I have used the alum enema my percentage of mortality in abdominal work has been a little less than one-half of what it was during the previous seven years. A portion of this decrease is doubtless due to improved methods of operating which have been introduced during that time. Another portion is doubtless due to the greater skill which comes to every man as his experience increases. But there still remains a wide margin which, in my judgment, may be reasonably attributed to the effect of the alum enema in eliminating intestinal paresis as a cause of death.

The use of the alum enema has not been confined to my own practice. Other surgeons who have seen its beneficial effects in my hands have adopted it with equally good results. It is now in common use in the Grady Hospital, St. Joseph's Infirmary, and other institutions in Atlanta. Nor is its usefulness limited to abdominal surgery. I often employ it after minor operations in patients who cannot retain a cathartic taken into the stomach, or when cathartics, though retained, fail to act. A number of the general practitioners of my own city have informed me that they have also used it in non-surgical cases, especially typhoid fever, with great satisfaction. It has, therefore, a wide range of usefulness, and it appears to me to constitute a valuable addition to the therapeutic resources of the profession.—*The American Journal of Obstetrics and Diseases of Women and Children.*

---

### SUCCESS IN SEXUAL THERAPEUTICS.

Probably no one class of cases so frequently and so generously contributes to fill the coffers of the irregulars whom the regular profession vainly essays to circumvent by legal enactment, as that of sexual weakness or decline of sexual power. Seminal emissions of the nocturnal variety are held up to weak and erring youth and adolescence as the forerunner of impotence and mental blight. Not alone are word pictures of the victims given, but wood-cuts, fearfully and wonderfully made, depict the awful state reached by those who have failed to embrace the means offered at the repair shop of the cure-all.

Regarding this matter of nocturnal seminal emissions the regular profession, it seems to me, has erred in its treatment of these subjects. In general the tendency of advice has been that such losses amount to very little and, therefore, are to be largely ignored. This bit of advice is not true, and it has served to send patients in droves to irregulars and mountebanks. The truth is that the material loss of semen amounts to very little, but the effect on the nervous system and the mental inquietude amounts to a great deal, and they always call for and deserve the attention which means relief without injury. It has long been a common report coming from the regular counsellor that losses are of no concern unless very frequent, and marriage or hard work would be all that was needed. Hard work meant the exhausting of the physical body so that the work of repair would keep mother Nature too busy to permit of wastes, while marriage presumably would change the nature of the loss. This advice is faulty because in neither case is there a correction of the cause of the trouble. Hard work can't continue always, and the peace bought at the price of exhaustion puts a ban upon pleasure since its time never comes. Correcting seminal losses by marriage offers a parentage of weakness, and from it a nation of weaklings must come. In addition, the union of sexual misfits keeps the divorce courts busy.

After all it is not the major operations and conduct of long-drawn out cases like typhoid fever that most concern the welfare of the race. For example, how often has as simple a matter as a case of chordee taxed one's patience and skill to direct easily. Modern methods have discarded the gatling-gun means, and now he who has become acquainted with the good points of black willow in suitable combination looks upon chordee as a mere passing epoch in a disease that has hardly enough such attributes to make it feared as it ought to be. I well remember how more than 25 years ago a Hebrew of most impulsive nature contracted a case of gonorrhea with strong chordeeic attachment. His medical advisor gave him enough bromide to cure an asylum of epileptics, and yet night after night an urgent appeal was made for help. Nowadays such a case could only miss sleep from ignorance of the doctor or failure of the patient to take a few pills or tablets. It is of just such trifles as this and these that the monument of success is made.

Possibly one of the main reasons why failure has met so many attempts to correct the shortcomings of below par sexuals is that the treatment has been too much of a routine nature. Cases of sexual weakness vary most astonishingly. One individual can stand almost an unlimited amount of abuse, both natural and unnatural, while another shows early evidences of the shock which means mental inquietude and physical decrepitude. One case may need a long course of alterative-tonic treatment, while another needs a sedative course with all brakes on. During the course of some years of study and a little writing on this subject, I have been appealed to by dozens of physicans all over the country asking for a formula. This shows a lack of appreciation of the work in hand. Given a case of aggravated nocturnal losses. To simply stop the losses is neither curative nor effective. Losses mean something. They are a result of a cause, and until the cause is made out cure is far off.

A very large number of medical men see in the varicocele which accompanies more than 95 per cent. of these cases an indication for operative interference. To my mind the operations are largely unnecessary and the results rarely such as to offer encouraging hope. Destruction of the enlarged veins has absolutely nothing to do with the weakness. The weakness is not always in the fabricating power of the testicle so much as it is in the storing of the semen. As might be expected, the ability to rival the ram, billy goat or jackass is not perpetuated long in the subjects of varicocele, but the procreative power lasts long in these subjects when excesses are not allowed to occur. Very few cases of varicocele need more than a cold local bath and occasionally a suspensory. The theory that enlarged veins interfere with the active secretion of semen is not borne out by observation nor by theory, since blood reaches the parts by the arteries, and it is only in the removal of a superabundance of blood that stagnation occurs. The fact that eight men of ten met have more or less varicocele is proof enough that it is not a disease for the operative monomaniac to get out his instruments over.

The rapid increase of cases of men in middle and beyond the middle of life who have perpetually defective water-works shows that sexual disorders do not recover alone or by the head-in-the-sand theory of marriage. So long as sexual riot is being run a reckoning is due, and when these cases are treated as other weaknesses are met, the regular profession will have less reason to watch legislatures for fear the bars will be let down to the goats.

Nature's recuperative powers are most wonderful and in no way is this fact more forcibly shown than in the recovery that so often occurs in cases where the most terrific abuse has been indulged in. Given youth or the earlier years of manhood, a fair constitution, a willingness to follow directions, including a reasonably abstemious life and helpful treatment, and not one case in a hundred of sexual weakness should fail of restoration to a degree of power commensurate with a happy domestic life and an age of comfort. Nor to accomplish this is there necessity of any mysterious formulæ or wonderful combination of rare drugs. Indications met, corrections made and a clear conception of what the needs are, and the work is half done. Too often the patient is weaker in mind than in body, and then anything short of actual accomplishment falls under the ban. In no other branch or division of medical practice is there greater call for the giving of remedies with care and with brains.—*J. A. DeArmand, M.D., Davenport, Iowa, in Wisconsin Medical Recorder.*

---

### DANGERS FROM MILK.

In an article entitled "Conclusions Based upon Three Hundred and Thirty Outbreaks of Infectious Diseases Spread through the Milk Supply" (*American Journal of the Medical Sciences*, May, 1901), Dr. George M. Kober introduces his remarks by briefly setting forth the possible ways in which milk may be dangerous to health:

1. Sour milk or milk on the point of souring is liable to produce gastric and intestinal catarrhs of acute or chronic character; as an example we have cholera infantum. The causes of souring are lack of cleanliness and high temperature. The author quotes Bitter as claiming that milk is unfit for food which contains over 50,000 bacteria per cc.m. However, as the number of bacteria can be reduced to from 4,000 to 6,000 per cc.m. with care, the above figures would seem, in our opinion, to be far too large to willingly put up with.

2. Milk may be unfit for use and cause sickness because the animals are improperly fed or are being treated with the various potent therapeutic agents which are eliminated through the mammary gland. The writer states that symptons of poisoning from arsenic, copper, iodin, lead, mercury, tartar emetic, atropine, colchicum, croton oil, strychnine, veratrum viride, etc., have been thus occasioned.

3. Milk may be the product of a diseased animal. The author states that local diseases of the udder, as garget, which causes pseudo-diphtheria, as well as general diseases, may be thus conveyed to the consumer. Among general diseases are "septic fevers, foot and mouth disease, cowpox, anthrax, pleuro-pneumonia, rabies and tetanus."

4. Tuberculosis he considers to be so frequently acquired from milk as to deserve a separate section.

5. Finally, but by no means least important, milk may acquire specific infective properties after it leaves the udder of the animal. The epidemics discussed in the paper are chiefly epidemics of typhoid, scarlet fever and diphtheria.

To the above headings might be added sophistication with chemicals; those most used at the present time are borax, boracic acid and formalin. Their dangers were discussed some time since in this department.—*Pediatrics.*

---

### AUTOMATIC SAFETY-VALVE STOPPER—A DEVICE PREVENTING THE BURSTING OF PEROXIDE OF HYDROGEN BOTTLES.

The great trouble with peroxide preparations is that if the containers are tightly corked, the oxygen which separates and is set free, slowly but constantly as time passes, accumulates, until the bottles can no longer stand the pressure and burst, or the corks are driven out. Of the two alternatives, the bursting of the bottles is the most objectionable feature on account of the danger attached to it.

a

(*a*) Puncture.

Cut No. 1. Illustrates the cross section of the safety valve rubber cork, showing the wooden top and the puncture at the bottom. A thin strip of paraffined paper is inserted into the puncture,

Containers of the hydrogen peroxide, U. S. P., which is a comparatively weak solution of $H_2O_2$, yielding but 10 volumes of oxygen, may be closed with a wooden stopper, which, by the porous nature of the material, permits the escape of the gas almost as soon as it is set free, thus avoiding explosion and rupture of the bottles or the driving out of the corks.

While these wooden stoppers answer very well for solutions of $H_2O_2$ responding to 10 volumes of oxygen or less, with stronger solutions, such, for instance, as Marchand's peroxide of hydrogen medicinal (15 volumes), or his hydrozone (30 volumes of oxygen) they are quickly attacked by the solutions, as are also the ordinary corks, and within four months are completely oxidized, not merely bleached, but rendered so soft that they cut like pot cheese. From that time the goods are unfit for sale.

(a) Puncture.

Cut No. 2. Illustrates the cross section of a bottle corked and capped with vegetable parchment and paraffined muslin; no wire.

In order to prevent these difficulties, and especially to obviate the bursting of the bottles containing hydrozone, Mr. Marchand, the manufacturer of that article and other well-known brands of peroxide of hydrogen, has devised an ingenious stopper which he calls the "automatic safety-valve rubber cork," and which is shown in the illustration.

The material of the stopper is vulcanized rubber. The beveled end is punctured through in such a manner that when the pressure in the bottle rises above 5 to 8 pounds to the square inch (according to the thickness of the rubber at the bottom, which may vary slightly), the excess of free oxygen finds free egress and thus relieves the tension.

Cut No. 3. Illustrates the top of the bottle with the seal.

This device is first inserted, and a plug of porous wood is then driven in, thus stiffening the rubber and completing the operation of "corking."

The capping consists of vegetable parchment covered with paraffined muslin, no wiring being used or needed.

It is easily seen that this style of closing the bottle obviates the possibility of bursting. Assuming even, that through some imperfection of the stopper, the puncture should close, as soon as the pressure rises to a point far within that required for rupture of the bottle, the stopper, not being wired down, will yield and be forced out.

Retail druggists who have for so many years been the chief sufferers and losers from the bursting of the peroxide containers, and the deterioration of the substance otherwise from the causes indicated above, will welcome Mr. Marchand's invention as a happy solution of what has to them been a very serious problem in the past, since it will enable them to supply their trade with the higher solutions of hydrogen peroxide, and especially that preparation of Marchand's, for which the stopper was particularly designed, "hydrozoned," which carries 30 volumes of ovygen.

The device described above—the automatic safety-valve stopper—having entirely obviated the danger arising from the explosion of bottles in handling, there is certain to be a largely increased demand for Marchand's concentrated solutions of the peroxide of hydrogen (which alone will be corked with the patented stopper), since physicians anxious to obtain quick results will never prescribe anything but the most active solutions, or those richest in active oxygen, and since druggists will be protected absolutely against loss by deterioration or explosion. The medical profession is being thoroughly advised of Mr. Marchand's new method of closing his bottles of "peroxide of hydrogen medicinal" and "hydrozone," and will be certain to avail themselves of the advantages thus guaranteed them.—*April, 1901, issue of the National Druggist of St. Louis.*

NOTE.—Remember there is no popping when corks are removed.

---

### THE AMERICAN MEDICAL ASSOCIATION AT ST. PAUL.

The meeting of the American Medical Association, held at St. Paul, June 4-7, was one that will mark a distinct epoch in the history of the organization by reason of the adoption of the revised constitution and by-laws, as prepared by the committee on revision appointed at the meeting at Atlantic City.

Under this constitution and by-laws the working basis of the association is entirely changed. The association now consists of delegates, permanent members, members by invitation, honorary members and associate members. Permanent members shall consist of members of State or county societies, on presentation of proper credentials. Members by invitation shall consist of distinguished physicians of foreign countries, who may be invited by the officers of sections, or of the association. Honorary members shall be physicians of foreign countries who have risen to preeminence in the profession of medicine. Associate members may consist of representative teachers and students of allied sciences, not physicians.

The executive business of the association will be transacted by the House of Delegates, which shall at no time consist of more than one hundred and fifty members.—This House of Delegates shall consist of (1) delegates elected by permanently organized state and territorial medical societies in affiliation with the association; (2) two delegates elected by each of the component Sections of this association; (3) one delegate each from the medical departments of the U. S. Army and U. S. Navy, and one from the U. S. Marine-Hospital Service. Each state and territorial society entitled to representation shall have the privilege of sending to the association one delegate for every 500 of its resident regular members, and one for any additional fraction of that number; but each affiliated state and territorial society shall be entitled to at least one delegate.

To carry out this plan it will become necessary to rearrange the state societies, so that the entire membership of county societies will represent the state society, which in turn necessarily will be required to create an executive body for the transaction of its official business.

This action, separating the business from the scientific interests of the American Medical Association, will, we believe, meet with general approval. Under the new rules, the business can be disposed of more expeditiously, and to better advantage of the association, and much time will be gained for the consideration of the scientific interests.

Contrary to general expectation the meeting was quite well attended, the register showing the presence of 1806 members, a falling off of 213, of the meeting at Atlantic City, the best attended one of any meeting of the organization.

The officers elected for the following year are: president, Dr. John A. Wyeth, New York; first vice-president, Alonzo Garcelon, Maine; second vice-president, A. J. Stone, Minnesota; third vice-president, A. F. Jonas, Nebraska; fourth vice-president, John R. Dibrell, Arkansas; treasurer, Henry P. Newman, Illinois; secretary, Geo. H. Simmons, Illinois; librarian, Geo. W. Webster, Illinois; Board of Trustees, term expiring 1904: W. W. Grant,

Colorado; John F. Fulton, Minnesota; T. J. Happel, Tennessee. Judicial Council: Geo. Cook, New Hampshire; H. H. Grant, Kentucky; John B. Murphy, Illinois; Philip Marvel, New Jersey; Louis H. Taylor, Pennsylvania; John L. Dawson, South Carolina; N. Fred Essig, Washington.

The appointments to deliver the annual addresses are as follows: Oration in Surgery, Harry Sherman, California. Oration in Medicine, Frank Billings, Illinois. Oration in State Medicine, J. M. Emmert, Iowa. Saratoga Springs, N. Y., was chosen as the next place of meeting, a selection which in view of the many advantages possessed by that city, will prove to have been a wise one.—*Penn. Med. Journal.*

---

### LEPROSY IN THE UNITED STATES.

Investigations are being directed from Washington to ascertain the number of cases of leprosy in the United States. The object of this investigation is to secure data with which to go before Congress and ask for the establishment of two leper hospitals in the United States, one in the North and one in the South. Up to this writing some 8,000 communications have been sent out from Washington, to which only about 2,000 replies have been received. These 2,000 replies show 275 cases of leprosy in the United States. What the remaining 6,000 letters of inquiry may reveal is yet to be seen. Quite a sufficient number of cases are already located to justify Congress in the establishment of two hospitals, one in the North and one in the South. These cases are so widely distributed that to my mind the general government is the proper one to take action in the matter. The reports to date show 74 cases in New Orleans, chiefly among the Italian population. In Minnesota there are 23 cases, mostly among Scandinavians and in rural districts. North Dakota reports 15 cases and South Dakota 2; Chicago 5 and New York 6 cases.

It is proposed to go before Congress next winter with all the data that can be obtained and ask Congress to establish these hospitals as stated above. It is said the disease is spreading rapidly in New Orleans, but in the Dakotas the patients are all in rural districts and well isolated, consequently the disease does not spread so rapidly.

The question is one that appeals to every thoughtful citizen, and if the physicians in this country will write to their immediate members in Congress at the proper time there will be little doubt about the passage of the bill establishing the hospitals. Now is the time to take the matter in hand and push it to a successful termination.—*Miss. Med. Record.*

## Figures and Tables

**Cut No. 1. f1:**
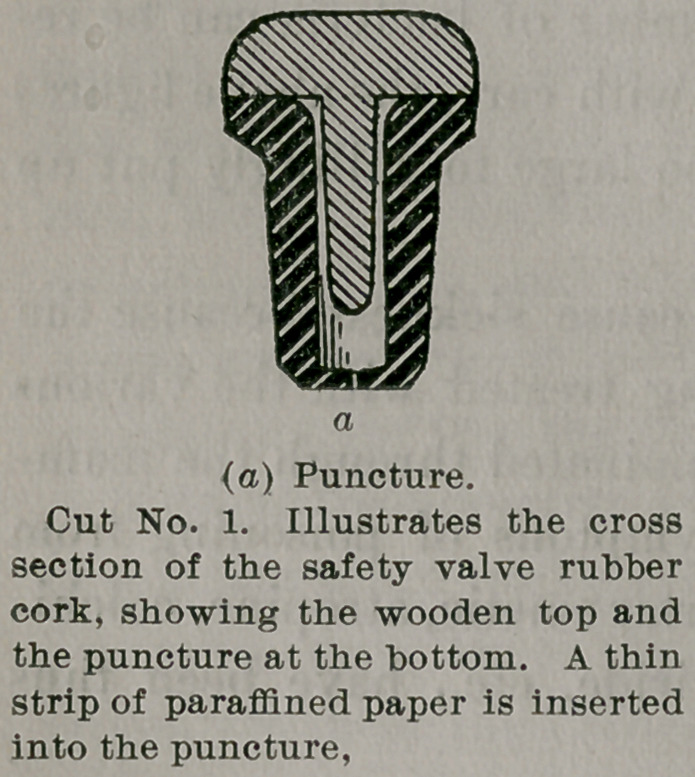


**Cut No. 2. f2:**
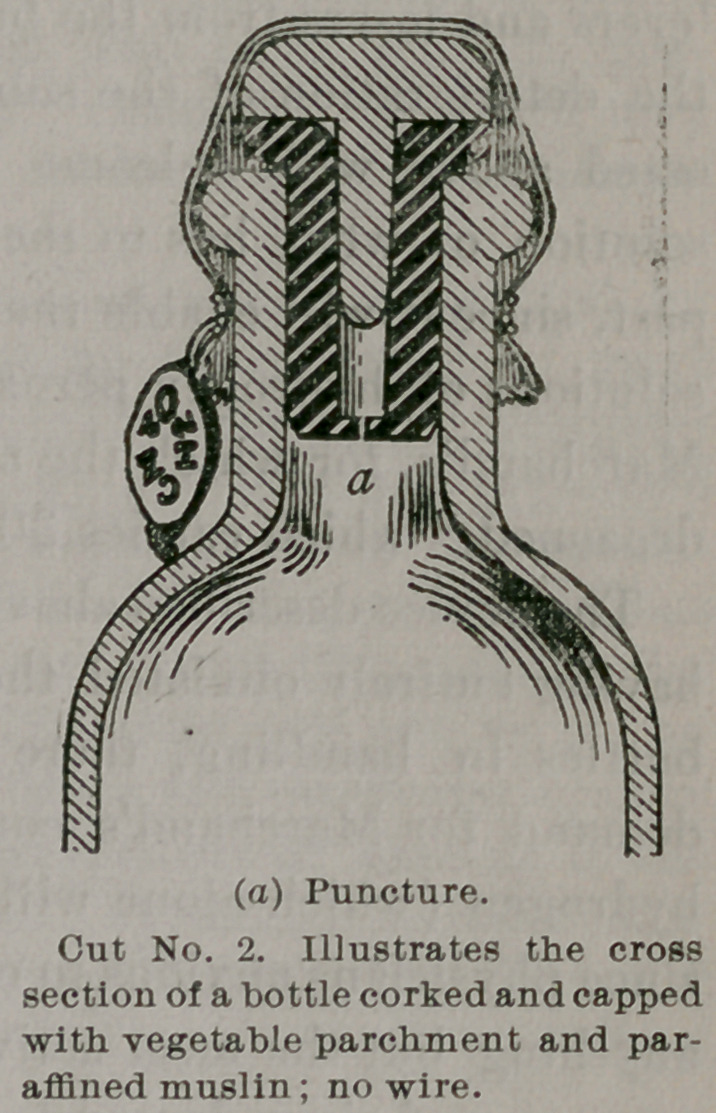


**Cut No. 3. f3:**